# Adrenergic receptor signaling regulates the CD40-receptor mediated anti-tumor immunity

**DOI:** 10.3389/fimmu.2023.1141712

**Published:** 2023-03-15

**Authors:** Akansha Singh, Ashish Ranjan

**Affiliations:** Department of Physiological Sciences, College of Veterinary Medicine, Oklahoma State University, Stillwater, OK, United States

**Keywords:** immunotherapy, Anti-CD40 agonist antibody, propranalol, adrenergic signaling, anti-tumor immunity

## Abstract

**Introduction:**

Anti-CD40 agonistic antibody (αCD40), an activator of dendritic cells (DC) can enhance antigen presentation and activate cytotoxic T-cells against poorly immunogenic tumors. However, cancer immunotherapy trials also suggest that αCD40 is only moderately effective in patients, falling short of achieving clinical success. Identifying factors that decrease αCD40 immune-stimulating effects can aid the translation of this agent to clinical reality.

**Method/Results:**

Here, we reveal that β-adrenergic signaling on DCs directly interferes with αCD40 efficacy in immunologically cold head and neck tumor model. We discovered that β-2 adrenergic receptor (β2AR) activation rewires CD40 signaling in DCs by directly inhibiting the phosphorylation of IκBα and indirectly by upregulating levels of phosphorylated-cAMP response element-binding protein (pCREB). Importantly, the addition of propranolol, a pan β-Blocker reprograms the CD40 pathways, inducing superior tumor regressions, increased infiltration of cytotoxic T-cells, and a reduced burden of regulatory T-cells in tumors compared to monotherapy.

**Conclusion:**

Our study highlights an important mechanistic link between stress-induced β2AR signaling and reduced αCD40 efficacy in cold tumors, providing a new combinatorial approach to improve clinical outcomes in patients.

## 1 Introduction

The unique ability of dendritic cells (DCs) to cross-present antigens to CD8+ T-cells makes them the most potent antigen-presenting cells (APCs) in anti-tumor immunity cascade. This is highly promising, but DCs with dysregulated CD receptor signaling fail to respond significantly to tumor antigens, resulting in poor antigen presentation and T-cell mediated tumor clearance. To overcome this challenge, the use of anti-CD40 agonistic antibody (αCD40), an activator of APCs has grown with the goal of enhancing the proportions of functional/activated DCs and subsequent activation of cytotoxic T-cells (1, 2). However, immunologically cold tumors can generate redundant immune evasive mechanisms to inhibit αCD40 immune activation by released tumor antigens, and clinical trials have shown that this approach is moderately effective as a monotherapy (3). Also, αCD40’s short circulatory half-life and toxicity can further limit its clinical utility (4–6). Thus, therapeutic approaches that increase sensitivity to αCD40 immunotherapy and thereby reduce the required treatment doses are needed to improve outcomes in patients with cold tumors. Herein, we aimed to dissect the role of β2-adrenergic signaling in αCD40 treatment, thereby providing a mechanistic and pharmacological basis to improve outcomes in clinical settings.

Adrenergic signaling mediated stress and anti-tumor immunity are intricately linked and demonstrate an inverse relationship. The sympathetic nervous system is closely associated with the body’s immune system since both primary and secondary lymphoid organs are permeated by post-ganglionic sympathetic neurons (7). The neurotransmitters (norepinephrine or NE) released from adrenergic neurons during stress can bind to the β2-adrenergic receptor (β2AR) present on tumor cell membranes. This binding activates anti-apoptotic pathways *via* adenylyl cyclase to induce rapid tumor growth rates, metastasis, chemo- and radio-resistance (8–11). The released NE also engages with macrophages, DCs, or T-cells to enhance macrophage polarization from M1 to M2 type, increases the production of anti-inflammatory cytokines, and reduces the proliferative capacities of cytotoxic T-cells (8, 12). Several studies have already demonstrated that β-ARs expressed on DCs decline pro-inflammatory cytokine secretion (13–16), antigen uptake (17), antigen presentation (18, 19), and migration capabilities (15, 20). What is not known is how the complementary activation mechanisms of αCD40 and β2AR influence DC effector functions, and whether targeting these signaling pathways concurrently would translate into superior tumor control relative to monotherapies.

Among the β2AR inhibitors, Propranolol, an FDA approved Pan-Beta blocker has been shown to improve outcomes of radiation (21), Immune checkpoint inhibitors (ICI) (22–24) & chemotherapies (25) in pre-clinical tumor models. Mechanistically, propranolol remodels tumor microenvironment by increasing the infiltration of effector CD8+ T-cells and declining suppressor cell populations. Propranolol hydrochloride is also being investigated in clinical trials as supportive therapy for prostate cancer (26) and stage IIIC-IV melanoma, and as part of combinatorial regimens of recurrent or metastatic urothelial cancer with anti-PD1 ICI (27), and radiation therapy for esophageal cancer (28). Based on these promising features, the aims of this study were two-fold. First, we assessed the implications of β-adrenergic signaling on DCs activation and maturation mediated by αCD40. Next, we evaluated the ability of propranolol to improve local αCD40 *in-situ* immunotherapy of poorly immunogenic head and neck tumors (MOC2). *In-situ* therapies utilize direct injection of immunostimulatory reagents into tumors to disrupt local immunosuppression, thereby reducing the dosage and associated toxicities. Our data shows that blocking β2AR can enhance the *in-situ* αCD40 efficacy against the MOC2 tumor model at suboptimal doses, thereby providing a translation basis of this approach for clinical use.

## 2 Materials

RPMI media (11875093), DMEM (11965092), Fetal bovine serum, FBS (10082147), Penicillin-Streptomycin, PenStrep (15140122), PBS (10010023), Collagenase IV (17104019), Pierce BCA Protein Assay (23228) were procured from ThermoFisher/Gibco, Waltham, MA, USA. Murine GM-CSF (315–03) was purchased from PeproTech, Cranbury, NJ, USA. Isoproterenol-HCL (16504), BSA (A7030) was purchased from MiliporeSigma, St. Louis, MO, USA. αCD40 (FGk45) from BioXcell West lebanon, NH. APC-Cy7 anti-CD45 (557659), Pe-Cy7 anti-CD45 (552848), BB515 anti-MHC-II (565254), BV421 anti-CD40 (562846) were purchased from BD Biosciences, San Jose, CA, USA. PerCP anti-CD3 (100326), BV785 anti-CD4 (100453), PE-Cy7 anti-CD8 (100722), APC-Cy7 anti-CD11c (117324), FITC anti-MHC-II (107605), PE anti-CD86 (105008), and ELISA MAX™ Deluxe Set Mouse IL-6 (431304), IL1b (432604) and IL-12 (433604) was procured from BioLegend, San Diego, CA, USA. TRIzol Reagent (15596026), PE anti-GranzymeB (12–8898–82), AF488 anti-FOXP3 (53–5773–82), unconjugated GAPDH (AM4300) antibody and MTT (3-(4,5-Dimethylthiazol-2-yl)-2,5-Diphenyltetrazolium Bromide) (M6494) were purchased from Invitrogen, Waltham, MA, USA. Unconjugated Phospho-IkBα (2859S), Phospho-CREB (9198S), IkBα (4814T) and CREB (9197T) antibodies were purchased from Cell Signaling Technology, Inc. Danvers, MA, USA. iScript™ gDNA Clear cDNA Synthesis Kit (1725034), iTaq Universal SYBR Green Supermix (1725124) was purchased from Bio-Rad, Hercules, CA, USA. IL-6, IL-1b, IL-10, and GAPDH primers were ordered from IDT, Coralville, IA, USA. The Quantikine™ Mouse IL-10 (M1000B) immunoassay was procured from R&D Systems, Inc., Minneapolis, MN, USA. Mouse OSCC (MOC2) cell line (EWL002-FP) was purchased from Kerafast, Boston, MA, USA.

## 3 Methods

### 3.1 Generation of tumor cell lysate, and bone marrow-derived Dendritic cells (BMDCs) for cytotoxicity and signaling studies

The lysate was generated by sonicating 10,000 MOC2 cells using Branson Sonifier 450 and centrifuging the lysate to harvest whole or partially lysed cells. The protein content of the whole lysate was determined using a BCA assay kit.

**Figure 1:**
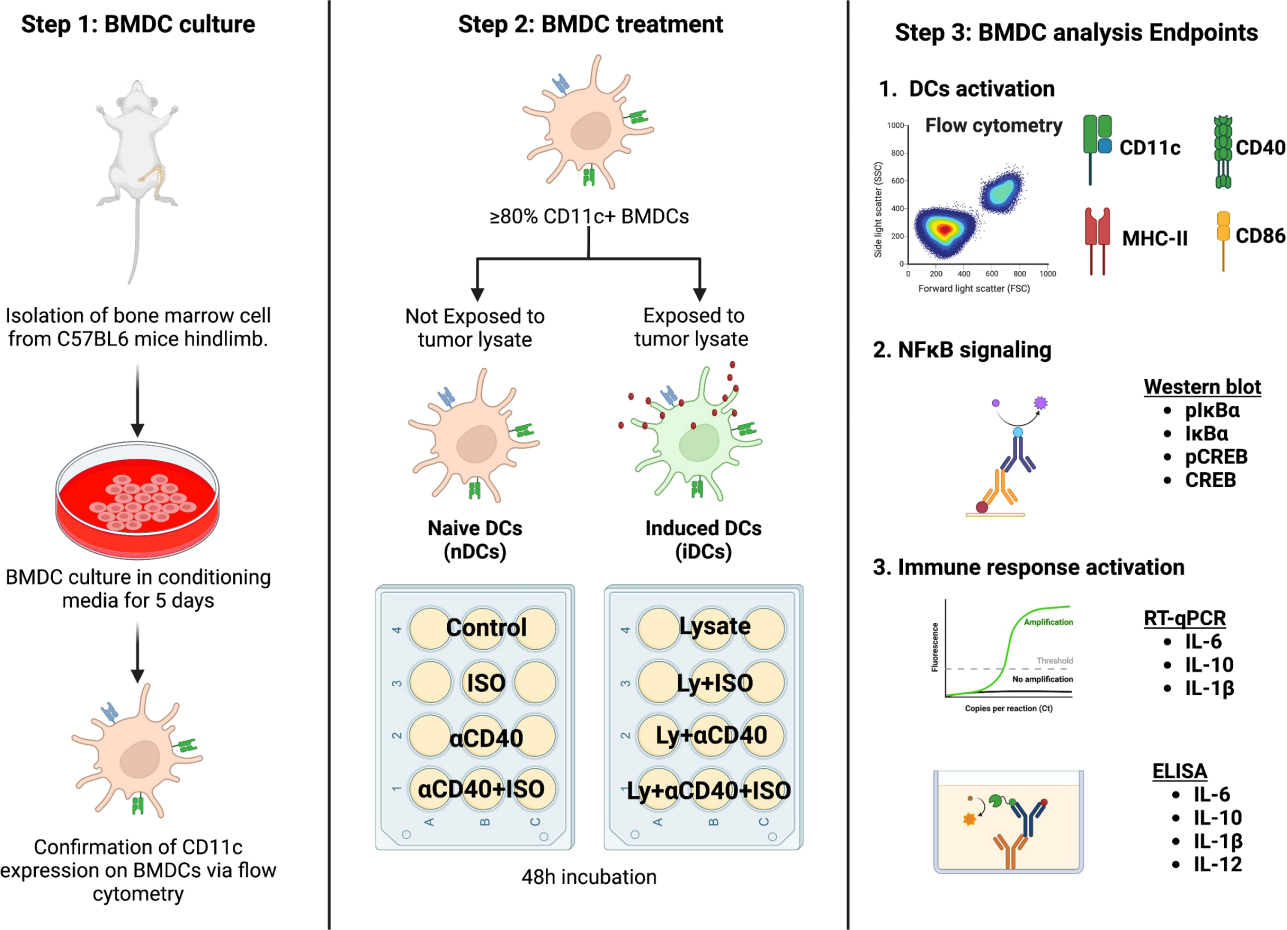
A schematic representation of the *in-vitro* BMDC isolation and treatment plan is shown. Bone marrow cells derived from C57BL/6 female mice were cultured in DC conditioning media and the expression of CD11c was confirmed after 5 days of culture. BMDCs with ≥80% CD11c expression were categorized as naïve (nDCs) or induced DCs (iDCs) based on exposure to MOC2 tumor cell lysate. The cells were then treated with 1 μM ISO (a β2AR agonist) and 10 μg/ml αCD40 (a CD40 agonist) for 48 hours before analysis. Schematic created with BioRender.com.

Female mice aged 8-12 weeks were utilized to generate bone marrow-derived DCs (BMDCs) through culturing of the harvested marrow cells with RPMI media that was enriched with FBS, Penicillin-Streptomycin, and GM-CSF. On the 5th day, the BMDCs were harvested, and their CD11c expression was confirmed using flow cytometry (≥80% CD11c+) and these cells were used for subsequent experiments as described in **Figure 1**. Briefly, the BMDCs were divided into two groups: naïve BMDCs (nDCs, untreated with tumor cell lysate) and induced DCs (iDCs, treated with tumor cell lysate). 25,000 BMDCs were treated with 1μM Isoproterenol (non-selective β2AR agonist, ISO), 10μg/ml αCD40 or 100 μg/ml tumor cells lysate and incubated for 48h in RPMI with 100ng/ml GM-CSF to assess cytotoxicity using the MTT assay (following manufacturer’s protocol), and for other mechanistic assays as described in following sections. For co-treatments, BMDCs were pretreated with 1μM ISO for 1h before being subjected to αCD40 or tumor cell lysate and incubated for 48h.

### 3.2 *In-vitro* and *in-vivo* immune cell analysis using flow cytometry

Briefly, iDCs and nDCs were washed with cold FACS buffer (PBS + 2% FBS) and stained for 30mins on ice in dark for CD11c, MHC-II, CD86 & CD40. Cells were washed twice with cold FACS buffer before acquisition on BD™ LSRII instrument. For *in-vivo* studies, MOC2 tumor samples from mice were cut into ~1cm pieces and digested with 200 U/ml Collagenase IV solution. Digested tumors were passed through 70 μm cell strainers and incubated in RBC lysis buffer (Invitrogen) for 10min. Single-cell suspensions were washed with cold FACS buffers and stained for 30mins on ice in dark. Data was analyzed using Flowjo software v.10.8.1 (Treestar Inc., Ashland, OR, USA) and cells were gated as follows- CD45+ CD3+ (Total T-cells), CD45+ CD3+ CD4+ CD8- (T_H_, CD4+ T helper cells), CD45+ CD3+ CD8- CD4+ FOXP3+ (Treg, Regulatory T-cells), CD45+ CD3+ CD4- CD8+ (T_C_, CD8+ T-cells), CD45+ CD3+ CD4- CD8+ GZMB+ (Effector cytotoxic T-cells), CD45+ CD11c+ (DC, Dendritic cells), CD45+ CD11c+ MHC-II+ CD86+ (Activated DCs), CD45+ CD11c+ MHC-II+ CD40+ (Matured DCs). Channel gating (**Figure S1**) and compensations were done using unstained cells, single-stained cells, and appropriate FMOs.

### 3.3 RT-qPCR analysis for cytokine gene expression in nDC and iDCs

RNA was extracted from treated and untreated nDCs and iDCs using TRIzol reagent followed by DNase treatment. cDNA was prepared from 1μg of total RNA using cDNA synthesis kit following manufacturer protocol. SYBR green based real-time analysis was done to detect the expression of IL-1β, IL-6 & IL-10 genes using GAPDH as a housekeeping gene. PCR mixture contained 1μl cDNA, 10μl SYBR green master mix (2X), and 100nM of each reverse and forward primer in a total volume of 20μl. PCR was run using settings of 95°C for 15s and 60°C for 60s for 35 cycles, on 7500 Fast Real-Time PCR system, Applied Biosystem. ΔC_T_ for the gene target was calculated by subtracting GAPDH C_T_ values for each replicate and represented as 2^ΔC_T_ in the bar graph. Primer sequences are given in **Table 1**.

**Table 1:** Mouse primer sequences used for RT-PCR.

Gene	5’ Primer sequence Forward	5’ Primer sequence Reverse
IL-1b	TGGACCTTCCAGGATGAGGACA	GTTCATCTCGGAGCCTGTAGTG
IL-6	ACAACCACGGCCTTCCCTACTT	CACGATTTCCCAGAGAACATGTG
IL-10	CGGGAAGACAATAACTGCACCC	CGGTTAGCAGTATGTTGTCCAGC
GAPDH	CATCACTGCCACCCAGAAGACTG	ATGCCAGTGAGCTTCCCGTTCAG

### 3.4 Western blot analysis of NFκB pathway targets and quantification of released cytokine

nDC and iDC were lysed and the total protein amount was estimated using BCA assay. An equal amount of cell lysate (30μg) was loaded on SDS-PAGE (BioRad, MiniPROTEAN Tetra System), and transferred to NC membrane (BioRad Trans-Blot Turbo) for 30min run at a constant voltage of 25V. Blots were blocked using 3% BSA solution for 1h at RT and stained with anti-pIkBα, anti-IkBα, anti-pCREB, and anti-CREB antibodies overnight at 4°C. Blots were washed in TBST buffer before incubating with appropriate secondary antibodies for 1h at RT, and imaged using BioRad, ChemiDoc™ MP Imaging System. Blots were stripped using mild stripping buffer (Glycine, pH2) and re-stained with anti-GAPDH antibody for 1h at RT following the above-mentioned procedure. To create intensity graphs, the intensities of pIkBα, IkBα, pCREB1, CREB, and GAPDH bands were measured using ImageJ software. The background intensities were subtracted from each blot. The intensities of pIkBα, IkBα, pCREB, or CREB were then normalized by dividing them with the GAPDH band intensities and represented as ratios of the phosphorylated to unphosphorylated IkBα or CREB on a bar graph. Data were analyzed using 2 independent BMDC experiments. ELISA was also performed to quantify the released cytokines, IL-6, IL-1β, IL-10 & IL-12, in 50μl of iDC culture supernatants following the manufacturer’s protocol.

### 3.5 MOC2 *in-vivo* study design

All animal associated procedures were approved by Oklahoma State University Animal Care and Use Committee. For tumor inoculation, MOC2 cells (purchased from Kerafast, Boston, MA, USA) cultured in DMEM media supplemented with 10% v/v FBS & 100U/ml PenStrep, harvested at 70-80% confluency were washed with sterile cold PBS before inoculations. 8-weeks old C57BL/6 female mice were injected subcutaneously with 1.5 X 10^5^ MOC2 cells in the flank region. Propranolol-HCl (10 mg/kg B.W.) resuspended in sterile PBS was injected subcutaneously from day 5 onwards daily until mice euthanasia. Tumor volumes were measured daily using a caliper (3-in Digital caliper, UltraTECH) and calculated using the formula (L*W*W)/2. Once tumors reached an average volume of ~50 mm^3^, 30 μg of αCD40 antibody was injected intratumorally. Two doses were given 8 days apart, and mice were euthanized 4-wk post-inoculation. Tumors were harvested for immune cell analysis using flow cytometry.

### 3.6 Statistical analysis

All analyses were performed using Prism v.9.4.0 software (GraphPad Software Inc. La Jolla, CA, USA). Treatment groups were compared using Two-tailed Unpaired T-tests. For group analysis with multiple variables, One-way ANOVA with either Tukey or Bonferroni multiple comparison test, and Two-way ANOVA were used as applicable. *P* values less than 0.05 was considered significant and represented as * *P <*0.05, ** *P <*0.005, *** *P <*0.0005, **** *P <*0.0001.

## 4 Results

### 4.1 β2AR signaling reduced the surface expression of co-stimulatory molecules on nDCs and iDCs.

**Figure 2:**
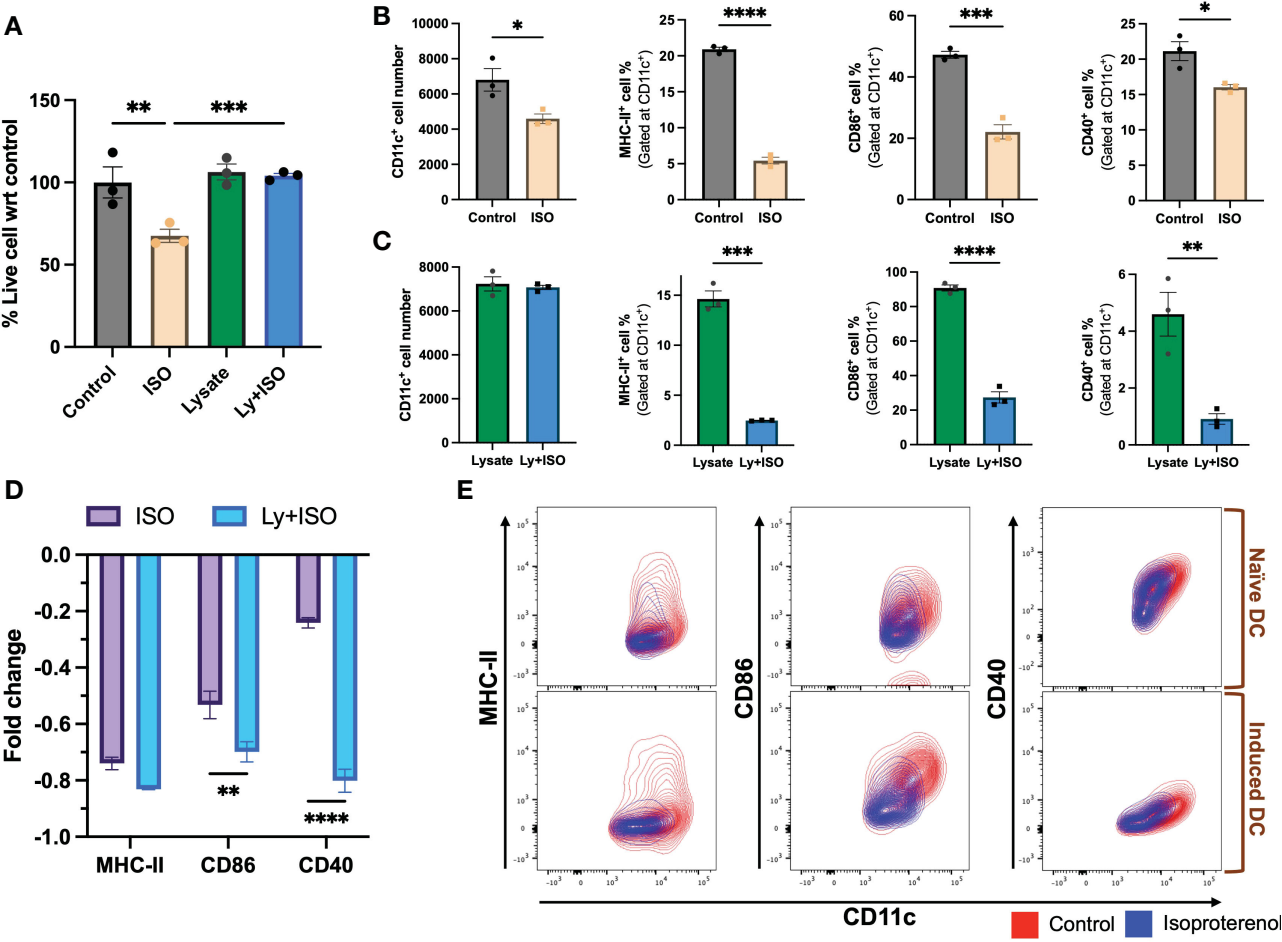
The impact of ISO (1µM) treatment on the viability and surface expression of co-stimulatory molecules on CD11c+ expressing naïve DC (nDCs) and tumor lysate induced DCs (iDCs) was evaluated after 48 hours. **(A)** ISO reduced nDCs viability, while having no effect on iDCs. **(B, C)** Both nDCs and iDCs treated with ISO showed significant decreases in surface expression of co-stimulatory molecules, MHC-II+, CD86+ and CD40+. **(D)** iDCs exposed to ISO showed a higher decrease in MHC-II, CD86 and CD40 surface expression compared to nDCs. The fold change was calculated by comparing the ISO treated population to the respective control (untreated nDCs or lysate-only treated iDCs) using the formula [(ISO treated population/Control population)-1]. **(E)** The results are demonstrated by representative contour plots of MHC-II+, CD86+ and CD40+ cell populations, showing the intensity of ISO treated nDCs and iDCs overlaid with their respective controls. Statistical analysis was carried out using unpaired t-test, One-way ANOVA & Two-way ANOVA tests where applicable. *P* values less than 0.05 were considered significant. * *P <*0.05, ** *P <*0.005, *** *P <*0.0005, **** *P <*0.0001.

β2AR agonist treated nDCs showed significantly reduced CD11c+ expression and viability (~40%) relative to untreated control, but these effects were not observed upon exposure to MOC2 tumor lysates in iDCs (**Figures 2A–C**). Also, the MHC-II, CD86 & CD40 expressions significantly decreased in both nDCs and iDCs with ISO (**Figures 2B, C, E** & **Figure S2**), but the average fold decrease was more pronounced in iDCs compared to nDCs (~MHC-II [83% vs 74%], CD86 [70% vs 53%] & CD40 [80% vs 24.1%]; **Figure 2D**).

### 4.2 β2AR signaling reduced αCD40 priming abilities of nDCs and iDCs.

**Figure 3:**
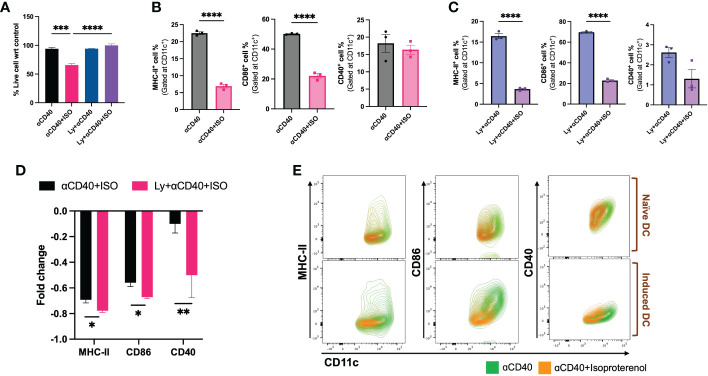
Viability & frequencies of MHC-II+, CD86+ & CD40+ nDCs and iDCs exposed to 10 µg/ml αCD40 and 1µM ISO for 48h. **(A)** A significant decrease in the viability of αCD40 treated nDCs with ISO treatment was observed in absence of tumor lysate stimulation. **(B, C).** The population of MHC-II+ and CD86+ nDC and iDC was reduced with ISO and αCD40 co-treatment, however, CD40+ population remain unchanged in nDCs but reduced in iDCs. **(D)** Decrease in the surface expression of co-stimulatory molecules was significantly higher in αCD40-treated iDCs compared to nDCs. Fold change in a cell population with ISO treatment was calculated using αCD40 only treated nDCs and iDCs as control and using the formula: [(ISO treated population/Control population)-1]. **(E)** The results are demonstrated by representative contour plots of MHC-II+, CD86+ and CD40+ cell populations, showing the intensity of ISO treated nDCs and iDCs overlaid with their respective αCD40 treatment controls. Statistical analysis was carried out using unpaired T-test, One-way ANOVA & Two-way ANOVA tests where applicable. *P* values less than 0.05 were considered significant. *** *P <*0.0005, **** *P <*0.0001.

Unlike iDCs, nDCs that were co-treated with αCD40 and ISO showed significantly reduced cell viability compared to αCD40 alone (**Figure 3A**). Additionally, although the MHC-II and CD86 expression on CD11c+ cells and percentage of CD40+ cell modified similarly for nDCs and iDCs (**Figure 3B, C** & **Figure S2**), however, the MFI of CD40 expression on iDC was significantly reduced relative to nDC (**Figure S2**), indicating an overall decline in the αCD40 mediated priming of iDCs following β2AR activation (**Figures 3D, E**).

### 4.3 β2AR signaling reengineered CD40 signaling by inhibiting phosphorylation of IκBα subunit of the Ikk complex to alter cytokine production.

**Figure 4:**
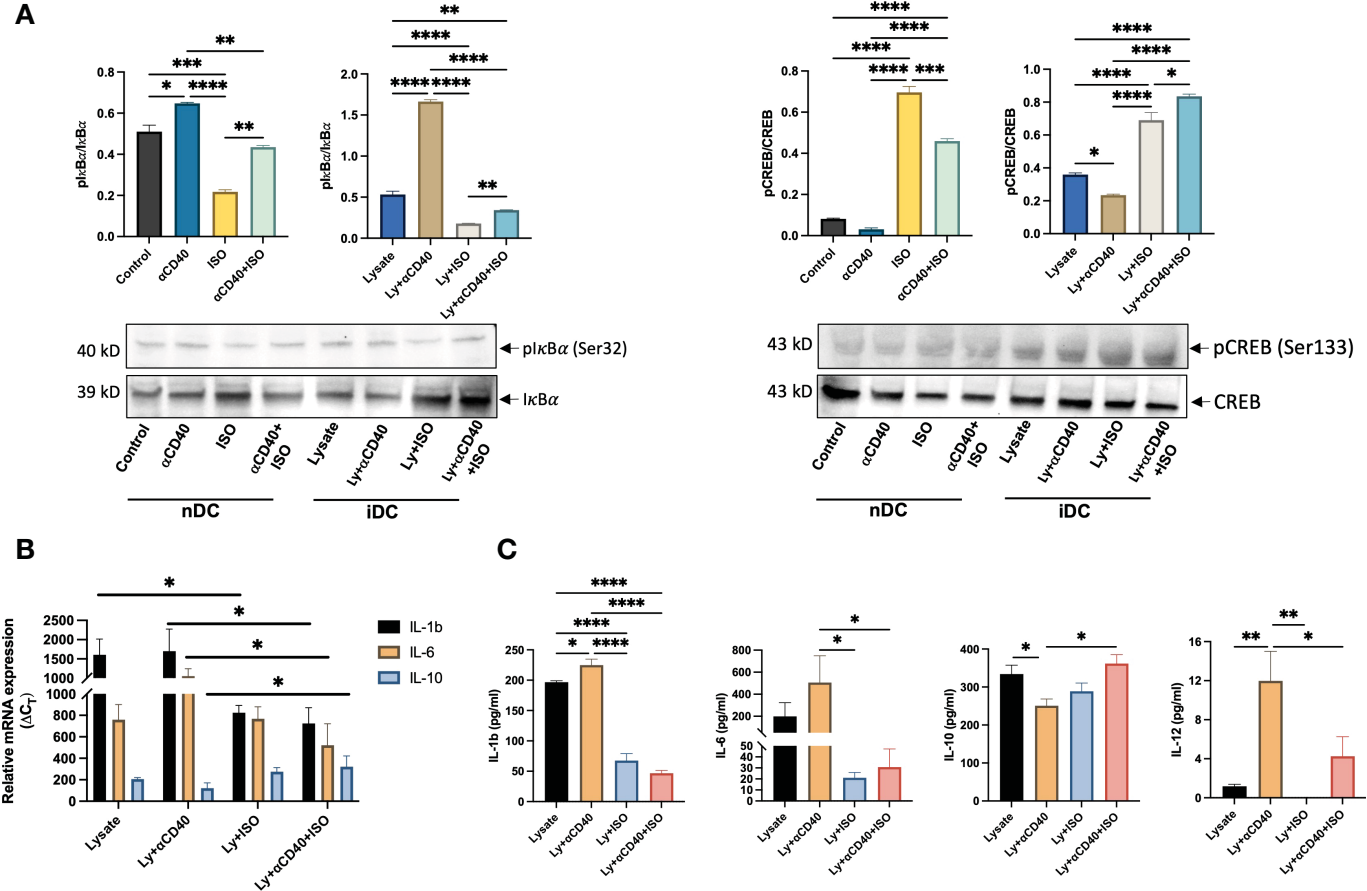
Analysis of Phosphorylated IkBα and CREB levels in nDCs and iDCs treated with ISO (1µM) and αCD40 (10 µg/ml) for 48h. **(A)** Western blots showed reduced pIkBα levels compared to unphosphorylated IkBα in the presence of ISO in both αCD40 treated nDCs and iDCs. ISO treatment increased pCREB levels in both nDCs and iDCs, with or without αCD40 treatment. The ratio of phosphorylated to unphosphorylated forms is shown as the intensity graphs. Respective GAPDH blots used to normalize the band intensities are shown in **Figures S4A, B** and the normalized band intensities are summarized in **Figure S5C**. **(B)** Gene expression analysis revealed that αCD40+ISO treatment significantly decreased IL-1β & IL-6 levels in iDCs compared to αCD40 treatment alone, and increased IL-10 expression in these cells. The results are expressed as 2^ΔCT with respect to GAPDH levels. **(C)** The release of IL-1β, IL-6 & IL-10 in the culture supernatant of co-treated iDCs showed a similar trend, with αCD40-mediated increase in released IL-12 significantly declining with ISO treatment. Statistical analysis was carried out using One-way ANOVA. *P* values less than 0.05 were considered significant. * *P <*0.05, ** *P <*0.005, *** *P <*0.0005, **** *P <*0.0001.

The activator of NFkB1 & NFkB2 complexes, i.e. IKK complex, was analyzed by targeting phosphorylated IkBα (pIkBα, Ser32) using western blot and represented as the ratio of phosphorylated and unphosphorylated IkBα band intensities. In both nDCs and iDCs, a significant increase in intracellular levels of pIkBα vs IkBα was observed with αCD40 treatment, but pIkBα levels declined significantly with αCD40+ISO cotreatment (~1.5-fold in nDCs and ~4-fold in iDCs). Also, the levels of phosphorylated CREB (pCREB, Ser133) relative to unphosphorylated CREB (CREB) decreased slightly in nDCs with αCD40 treatment, but this phenomenon was more evident in iDCs. The addition of ISO (± αCD40) significantly increased the levels of pCREB in both nDCs and iDCs (**Figure 4A**). To understand the association of IKK complex activation with cytokines production, the gene expression of IL-1b, IL-6 & IL-10 in iDCs was quantified. Expression of IL-10 decreased, and IL-1b & IL-6 increased in presence of αCD40 in iDCs. In contrast, ISO significantly decreased the expression of IL-1b and IL-6 and increased the expression of IL-10 in αCD40 treated iDCs. (**Figure 4B**). These were similarly observed in culture supernatant with significant decreases in pro-inflammatory cytokine levels of IL-1β, IL-6, and IL-12 and an increase in anti-inflammatory IL-10 levels in αCD40+ISO treated iDCs relative to αCD40 treated cells (**Figure 4C**).

### 4.4 αCD40 and propranolol combination achieved superior MOC2 tumor suppression and induction of anti-tumor immunity

**Figure 5:**
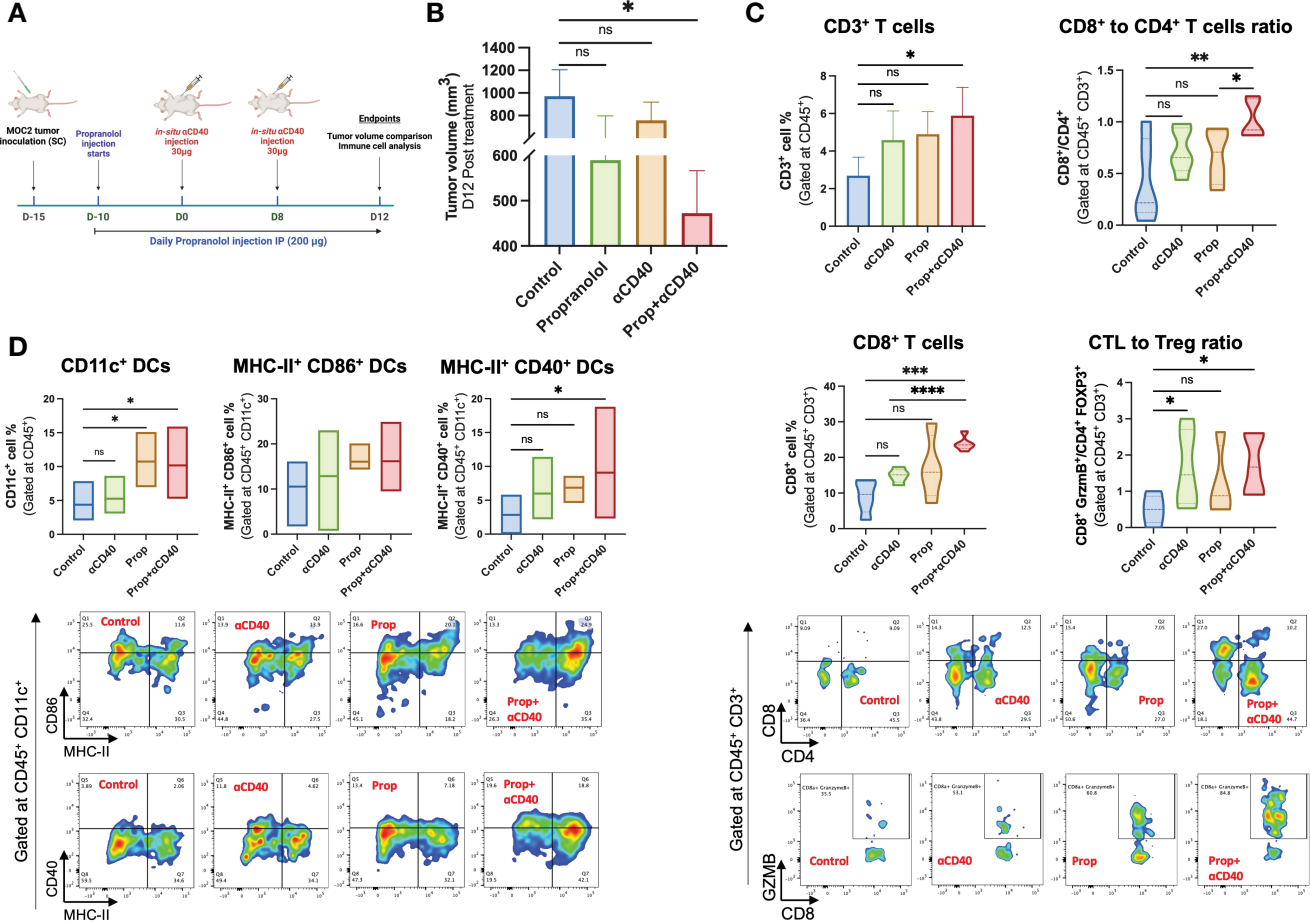
Treatment design of the murine efficacy and immune-evaluation study. **(A)** Propranolol (10mg/kg of BW) was administered subcutaneously daily 5 days post-inoculation. Two 30 µg αCD40 intratumoral injections were administered at 8 days intervals in the tumor (~50 mm^3^ volume). Mean tumor volume and anti-tumor immune cells were compared on day 28 post-inoculation (Timeline created with BioRender.com). **(B)** The combination of Prop+αCD40 demonstrated a significant reduction in tumor volume compared to the control on day 28 post-inoculation, while monotherapies did not show any significant differences. These results suggest that the combination therapy of Prop+αCD40 is more effective in reducing tumor growth compared to either Prop or αCD40 alone. Immune cells infiltrating MOC2 tumors (n=5 mice/group) analyzed by flow cytometry showed superior immunomodulation with Prop+αCD40. **(C)** Frequencies of CD3+ T-cells, especially CD8+ T-cells infiltrating tumors were enhanced at the highest level by combination treatment vs untreated control and monotherapies. The ratio of cytotoxic T-cells (CD8+ GZMB+) to T regulatory cells (CD4+ Foxp3+) was increased significantly in αCD40 treated groups relative to the control. **(D)** CD11c+, MHC-II+ CD86+ double positive (gated at CD45+ CD11c+) & MHC-II+ CD40+ double positive (gated at CD45+ CD11c+) dendritic cell frequencies showed significant enhancements in the presence of Prop and αCD40. Statistical analysis was carried out using One-way ANOVA & Two-way ANOVA multiple comparison tests. *P* values less than 0.05 were considered significant. * *P <*0.05, ** *P <*0.005, *** *P <*0.0005, **** *P <*0.0001. ns, nonsignificant.

Propranolol mediated efficacy of αCD40 *in-situ* vaccination (ISV) in the MOC2 model was evaluated by comparing tumor growth up to 4 weeks post-inoculation (see **Figure 5A** schematic). αCD40 and propranolol induced partial to moderate reduction of tumor growth compared to the untreated control, but the combined treatment achieved a significant suppression of tumor growth rates compared to the control (p<0.05; **Figure 5B**). We also evaluated the percentage of T-cells and their functional counterparts in the tumor. Data showed a significant increase in the frequency of tumor-infiltrating CD45+ CD3+ T-cells in the combination regimen compared to control which was not observed with monotherapies (**Figure 5C**). Notably, a significantly higher infiltration of CD8+ T-cells in Prop+αCD40 treated tumors (~24%) relative to untreated tumors (9%) and αCD40 (~15%) was noted. A significantly higher numbers of CD8+ T cells vs CD4+ T cells was observed with combination treatment compared to control and Prop alone treated tumors. A non-statistically significant decrease in regulatory T-cells (CD4+ Foxp3+) populations and an increase in cytotoxic T-cells (CD8+ GZMB+) was observed with all therapies compared to control but their ratio demonstrated a significantly higher number of cytotoxic T-cells vs Tregs in αCD40 and Prop+αCD40 groups compared to the untreated control. Further, the addition of Prop enhanced CD45+ CD11c+ cells by 2.5-fold in treated tumors compared to untreated and αCD40 treated tumors (**Figure 5D**). Additionally, the mean population of MHC-II & CD86 double-positive cells increased from ~11% in untreated tumors to ~13% in αCD40, and ~16% in Prop+αCD40 & Prop alone treatments. Importantly, the MHC-II+ CD40+ double-positive DCs in Prop+αCD40 treated tumors showed the highest enhancement (~3-fold, p<0.05) vs untreated tumors, and Prop and αCD40 monotherapies (~2-fold). (All immune cell population data are presented in **Supplementary Table 1**.)

## 5 Discussion

Prior research has shown that NE-mediated adrenergic receptor activation inhibits the activation, differentiation, and effector functions of T-cells (29–32) and modulates the cytokines and chemokines production from macrophages, monocytes, and DCs (7, 33, 34). β2AR signaling can also polarize macrophages from M1 to M2 type, thereby enhancing anti-inflammatory cytokines like IL-10, IL-4 & IL-13, and decreasing pro-inflammatory cytokines like INFγ, TNFα, IL1β, CCL2, CCL3 and CCL4 to cause tumor progression (35–39). Considering that CD40 pathways mainly impact APCs, the reported roles of β2AR signaling on macrophages and DCs piqued our interest in understanding their ability to modulate their functions and impact therapeutic outcomes. We found that β2AR signaling limits DCs function in the presence of tumor antigens by decreasing the expression of MHC-II, CD86, and CD40.

Our data disagrees with a previous study (40) that showed no effects on MHC-II & CD86 expression in NE and LPS stimulated DCs. We believe that unlike LPS that works *via* TLR4, DAMPs & PAMPs present in tumor cell lysate can induce DC activations through multiple signaling mechanisms (TLR3, TLR4, RAGE, etc.) on DCs (41, 42), thereby generating dramatic different immunoactivities with β2AR activation vs LPS alone. To demonstrate this, we compared the stimulation of BMDCs with two types of cancer cell lysates (B16F10 melanoma and MOC2 oral squamous carcinoma) to that of LPS treatments. Our results showed that LPS-treated BMDCs did not exhibit any changes in MHC-2 and CD86 surface expression, while BMDC stimulation with B16F10 and MOC2 tumor cell lysates resulted in differential MHC-2 expression in response to ISO treatment (as shown in **Figure S3**). While we did not investigate the specific mechanisms behind the differences in MHC-2 and CD86 expression on DCs with the various types of cell lysates, our data still provides strong evidence that the expression of immunomodulatory markers on DCs is dependent on the composition of the ligand/antigen pool.

**Figure 6:**
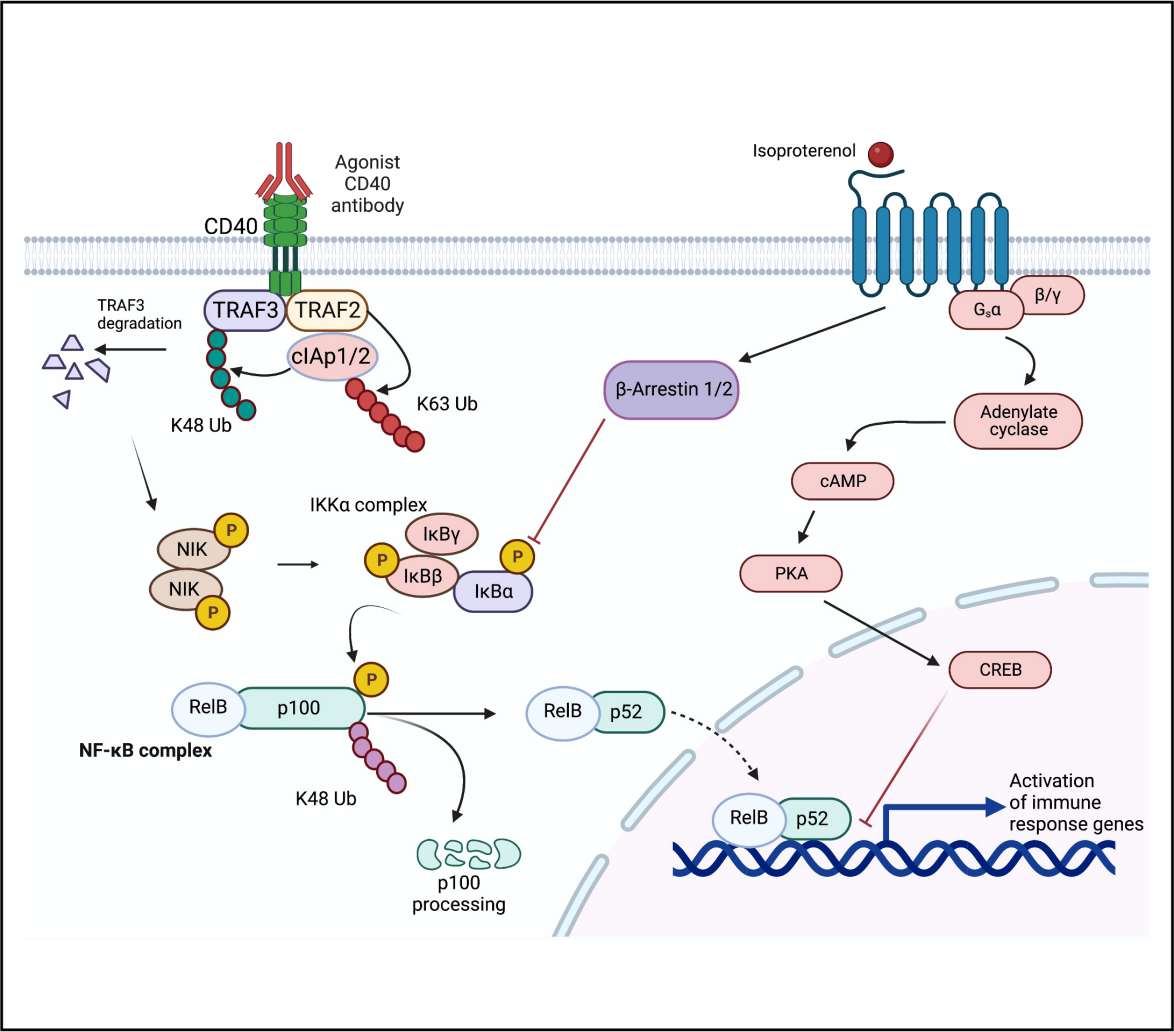
Proposed mechanism of β2AR signaling mediated re-engineering of CD40-CD40L signaling in DCs. An increase in intracellular pIkBα (pIkBα) level with αCD40 treatment is reversed by β2AR signaling, thereby resulting in an altered cytokine production and immuno-retardation of anti-tumor response. Adapted from “NF-KB Signaling Pathway”, by BioRender.com (2022). Retrieved from https://app.biorender.com/biorender-templates.

The use of Pan Beta-blocker like propranolol enhances the efficacy of immunotherapy by blocking β2AR signaling on progenitor and functional immune cells to result in better therapeutic outcomes (22, 27, 31). We looked at the effects of β2AR activation on DCs in presence of αCD40 in BMDCs. β2AR signaling can regulate NFκB pathways by inhibiting phosphorylation of IkBα through enhanced β-Arrestin2 protein production (43, 44). We found that activation of β2AR on αCD40 treated DCs significantly decreased the levels of pIkBα and enhanced the accumulation of unphosphorylated IkBα in cells, thereby suggesting the suppression of DC maturation and CD40-mediated NFκB activation. β2AR signaling has also been shown to activate cAMP/PKA pathways and subsequent CREB phosphorylation. We observed higher levels of pCREB in ISO treated nDCs and iDCs (**Figure 4A**). Phosphorylated (p) CREB can compete with activated NFκB for the DNA binding sites. Thus, we propose that pCREB may indirectly interfere with αCD40 mediated DC priming (see **Figure 6** schematic), to decrease the production of pro-inflammatory cytokines and co-stimulatory molecules (e.g. decreased IL-6 and IL-1B expression and enhanced IL-10 production in iDCs) (45, 46). We noticed similar trends in the cytokines produced in the culture supernatants. The ability of DCs to activate T cells is often evaluated by measuring IL-12 production and co-stimulatory molecules (47, 48), so we examined the IL-12 released from these cells and found that ISO treatment had a significant impact on the release of IL-12 from αCD40-treated induced DCs (iDCs). Thus, we propose that activation of β2AR signaling in αCD40-treated naïve and induced DCs transforms the DC population into an immune-tolerant type, both by blocking NFκB activation and indirectly promoting CREB activation.

Propranolol has been shown to improve the functions of naïve and activated immune cells (22, 27, 31). Since β2AR activation subverted αCD40 signaling in BMDCs, we also investigated the effects of pharmacological β2AR blocking on therapeutic outcomes of αCD40 in immunologically cold MOC2 tumors (49). Despite the improvements made in the potency of CD40 monoclonal antibodies (mAbs) through approaches such as engineering the Fc region, the use of αCD40 immunotherapy in clinics is still faced with challenges due to its associated toxicities. These toxicities include liver damage, low platelet count, cytokine release syndrome (CRS), and hyper-immune activation (2, 50, 51). Also, the widespread expression of the CD40 receptor on both immune and non-immune cells in tumors and other organs leads to broad activation of CD40-expressing cells, limiting the treatment dose of αCD40 mAbs and causing non-specific toxicity. Therefore, we employed a lower dose of αCD40 antibody (30µg; 2X) with Propranolol *via* intratumoral route to overcome the dose-limiting toxicities associated with higher quantities of αCD40 (4). β-blocker with αCD40 treatment significantly suppressed tumor growth at suboptimal doses compared to untreated control, and the outcomes were comparable to a prior report administering higher αCD40 dosage (50µg) and greater frequency (3X) of treatment in the melanoma model (52). MOC2 tumors show a high presence of Tregs and minimal populations of CD8 T-cells (53, 54). Blocking β2AR in mice tumor models can increase lymphocyte infiltration, and lower M1 to M2 polarization of macrophage and Treg population (22, 30, 55, 56). Our findings demonstrate that the combination of Prop+αCD40 effectively modulates the tumor microenvironment, leading to a pronounced increase in CD8+ T cell and cytotoxic T cell infiltration, and a reduction in Tregs compared to tumors treated with monotherapies or untreated control. Surface expression of MHC and co-stimulatory molecules on DCs are known to correlate with enhanced T cell activation and its effector functions (57–61). Blocking β2AR signaling with Prop significantly increased intratumoral CD11c+ populations, and when combined with αCD40 treatment, the number of double-positive MHC-II+ CD40+ DCs significantly increased. This suggests that the combination treatment facilitated robust anti-tumor immune cell priming and maturation. The increased presence of dendritic cells in the tumor microenvironment, facilitated by Prop treatment, provided a stronger foundation for αCD40 immune therapy. As a result, the combination treatment showed a significant improvement in adaptive anti-tumor immunity compared to control, unlike monotherapies. For *in-vivo* studies, our goal was to include a true untreated control to simulate clinical conditions and evaluate the immunological synergism between αCD40 & Propranolol. PBS is not a standard treatment for head and neck tumors and other types of tumors. Therefore, previous pre-clinical studies, including ours, have used untreated controls relative to PBS to assess the effectiveness of *in-situ* vaccination or parenteral treatment (52, 62–64). Additionally, studies by Hu et al., Singh et al., and others have shown that PBS or non-relevant/isotype control antibody treatments do not enhance tumor control compared to propranolol or αCD40 alone (64–68). Future studies may include PBS (or an isotype control antibody) to further confirm the feasibility of the proposed combination therapy in the MOC2 murine model.

Thus, our investigation provides the foundational basis for improving αCD40 immunotherapy by the use of β-blockers. This combination can be particularly relevant for cold TMEs and can reverse β2AR signaling mediated tumor cell survival seen previously (69, 70). We found that the combination of Prop+αCD40 enhanced therapeutic and anti-tumor immune responses compared to control, however, some changes were not statistically different from monotherapies. Further studies are needed to optimize treatment dosages and timelines to achieve a pronounced tumor remission. Gender-specific differences in β-blocker response and immune cell characteristics should also be explored to gain a deeper understanding of the therapeutic outcomes (71).

## Data availability statement

The original contributions presented in the study are included in the article/**Supplementary Material**. Further inquiries can be directed to the corresponding author.

## Ethics statement

The animal study was reviewed and approved by Oklahoma State University Animal Care and Use Committee.

## Author contributions

AR and AS conceived the project and designed the study goals. AS performed the experiments and analyzed the data. AR and AS wrote the manuscript. All authors contributed to the article and approved the submitted version.

## Supplementary material

Supplementary Figure 1 Gating strategy used for the analysis of BMDCs.

Supplementary Figure 2 Mean fluorescence intensities of different surface markers, MHC-II **(A)**, CD86 **(B)**, and CD40 **(C)** analyzed on CD11c gated BMDCs. Population percentage is shown in **Figure 2** and **Figure 3**. Statistical analysis was carried out using One-way ANOVA. *P* values less than 0.05 were considered significant. * *P <*0.05, *** *P <*0.0005, **** *P <*0.0001.

Supplementary Figure 3 MHC-II and CD86 expression on CD11c+ BMDCs under different stimulation. **(A)** 10,000 BMDC (>80% CD11c+) were stimulated with 1 µg/ml LPS and in combination with 10 µg/ml αCD40 for 48h showed a significant increase in MHC-II and CD86 expression. Upon ISO treatment, LPS alone stimulated BMDCs demonstrated no change in MHC-II and CD86 expression whereas it decreased in LPS+αCD40 treated BMDCs. **(B)** BMDCs treated with 100 µg/ml B16F10 tumor cell lysate showed a significant decrease in MHC-II expression which increased with the addition of αCD40. With ISO treatment, MHC-II expression further increased in these cells whereas CD86 expression decreased. **(C)** BMDCs treated with 100 µg/ml MOC2 tumor cell lysate alone and with αCD40 showed a similar pattern of MHC-II expression as B16F10 lysate stimulated cells but MHC-II and CD86 expression decreased upon ISO treatment. Statistical analysis was carried out using One-way ANOVA. *P* values less than 0.05 were considered significant. * *P <*0.05, ** *P <*0.005, *** *P <*0.0005, **** *P <*0.0001.

Supplementary Figure 4 Western blots of phosphorylated and unphosphorylated IkBα **(A)** & CREB **(B)**, presented in **Figure 4A**, are shown with their respective GAPDH blots used for normalization. **(C)** Table summarizing normalized band intensities of target proteins represented as a ratio of phosphorylated and unphosphorylated forms of IkBα & CREB in **Figure 4A**.

Supplementary Table 1 Frequencies of immune cells analyzed in murine MOC2 tumor model treated with β2AR antagonist (Prop) and CD40 agonist (αCD40).
