# Supplementary figures and images for: Adrenergic receptor signaling regulates the CD40-receptor mediated anti-tumor immunity
Source: Front Immunol. 2023 Mar 15;14:1141712. doi: 10.3389/fimmu.2023.1141712 (PMC10050348; doi:10.3389/fimmu.2023.1141712)

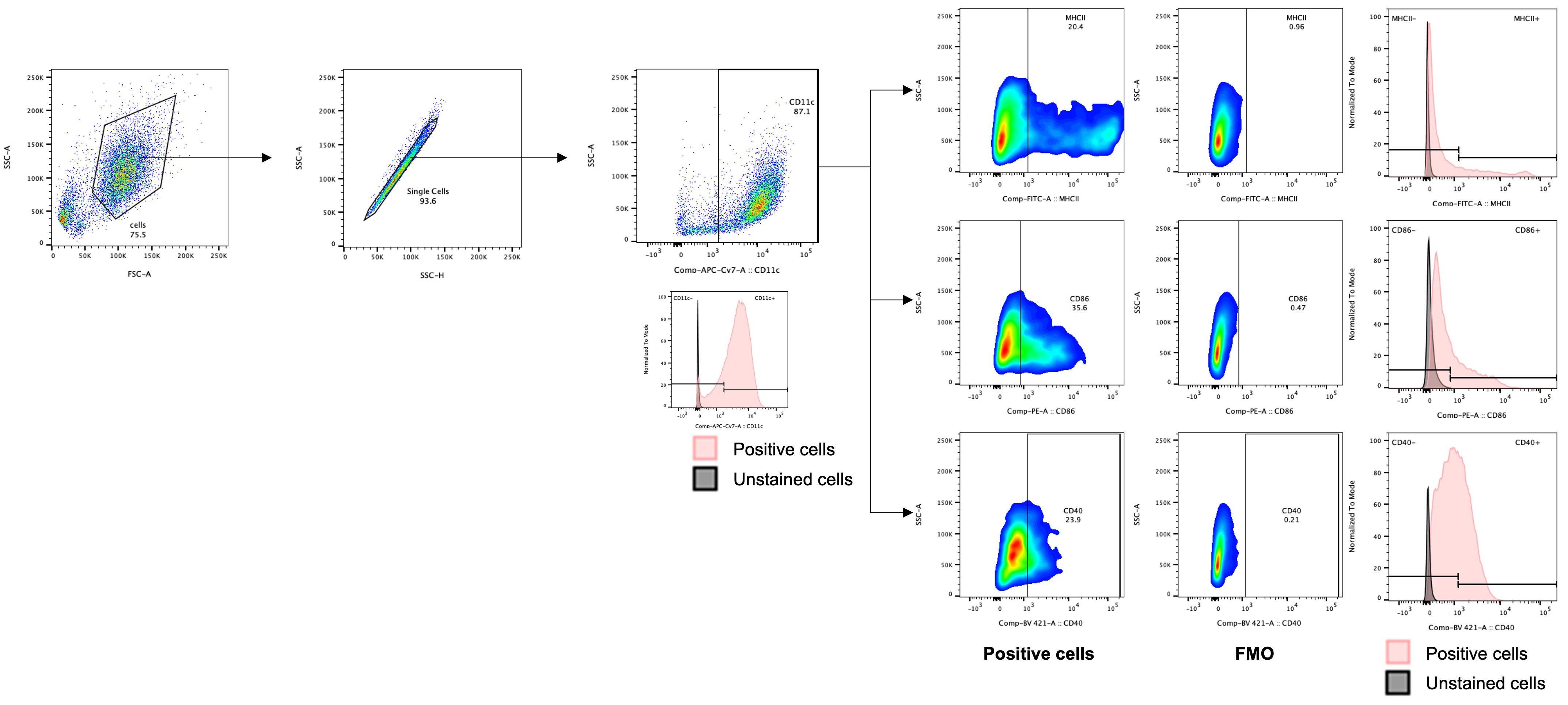

Supplement: Supplementary Figure 1 — Gating strategy used for the analysis of BMDCs. [file Image_1.jpeg]

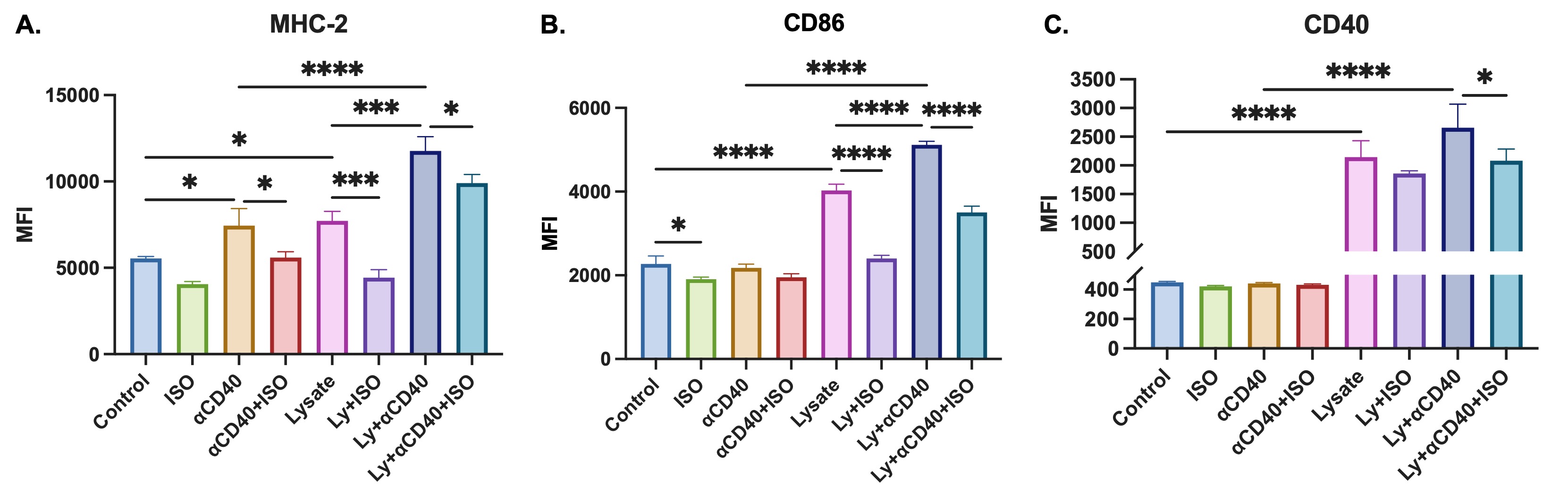

Supplement: Supplementary Figure 2 — Mean fluorescence intensities of different surface markers, MHC-II (A), CD86 (B), and CD40 (C) analyzed on CD11c gated BMDCs. Population percentage is shown in Figure 2 and Figure 3 . Statistical analysis was carried out using One-way ANOVA. P values less than 0.05 were considered significant. * P <0.05, *** P <0.0005, **** P <0.0001. [file Image_2.jpeg]

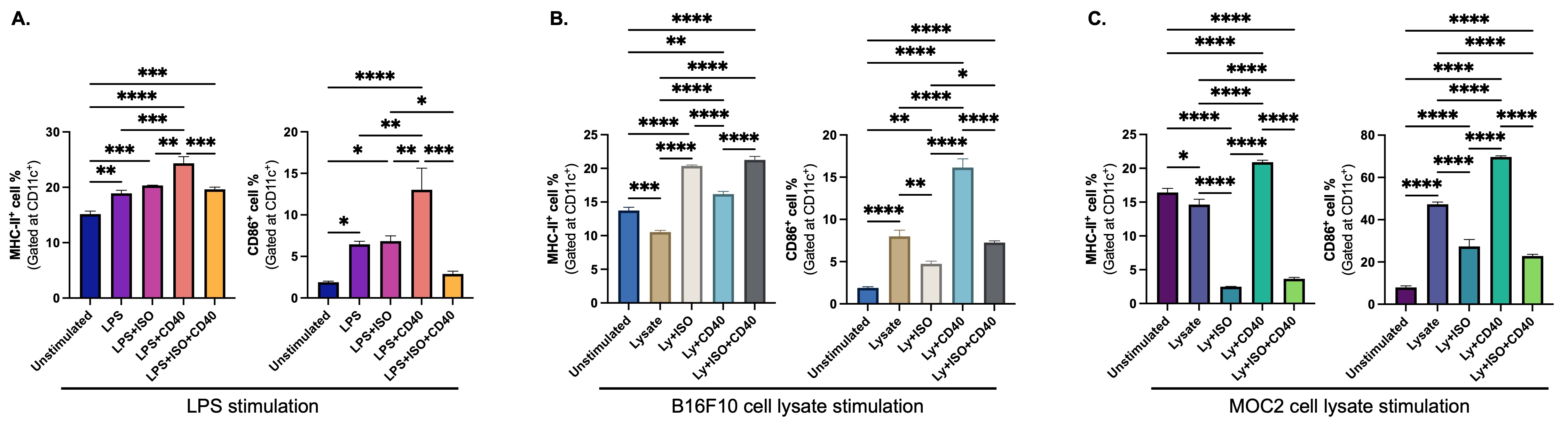

Supplement: Supplementary Figure 3 — MHC-II and CD86 expression on CD11c+ BMDCs under different stimulation. (A) 10,000 BMDC (>80% CD11c+) were stimulated with 1 µg/ml LPS and in combination with 10 µg/ml αCD40 for 48h showed a significant increase in MHC-II and CD86 expression. Upon ISO treatment, LPS alone stimulated BMDCs demonstrated no change in MHC-II and CD86 expression whereas it decreased in LPS+αCD40 treated BMDCs. (B) BMDCs treated with 100 µg/ml B16F10 tumor cell lysate showed a significant decrease in MHC-II expression which increased with the addition of αCD40. With ISO treatment, MHC-II expression further increased in these cells whereas CD86 expression decreased. (C) BMDCs treated with 100 µg/ml MOC2 tumor cell lysate alone and with αCD40 showed a similar pattern of MHC-II expression as B16F10 lysate stimulated cells but MHC-II and CD86 expression decreased upon ISO treatment. Statistical analysis was carried out using One-way ANOVA. P values less than 0.05 were considered significant. * P <0.05, ** P <0.005, *** P <0.0005, **** P <0.0001. [file Image_3.jpeg]

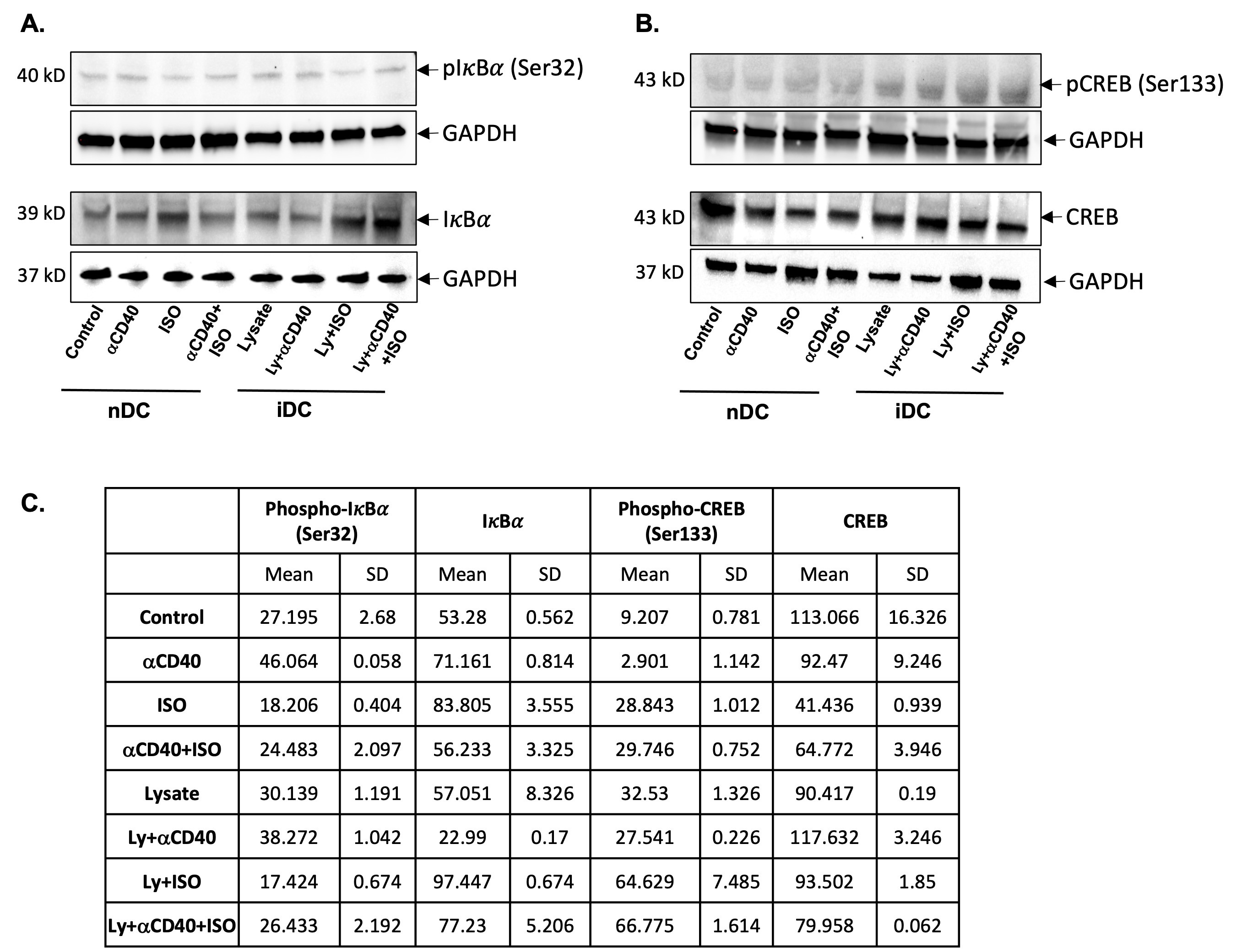

Supplement: Supplementary Figure 4 — Western blots of phosphorylated and unphosphorylated IkBα (A) & CREB (B), presented in Figure 4A , are shown with their respective GAPDH blots used for normalization. (C) Table summarizing normalized band intensities of target proteins represented as a ratio of phosphorylated and unphosphorylated forms of IkBα & CREB in Figure 4A . [file Image_4.jpeg]
